# Editorial: Insights in respiratory pharmacology: 2021

**DOI:** 10.3389/fphar.2022.1052994

**Published:** 2022-11-07

**Authors:** Paolo Montuschi, Irfan Rahman

**Affiliations:** ^1^ Faculty of Medicine, Imperial College, National Heart and Lung Institute, London, United Kingdom; ^2^ Department of Pharmacology, Faculty of Medicine, Catholic University of the Sacred Heart, Roma, Italy; ^3^ Department of Environmental Medicine, School of Medicine and Dentistry, University of Rochester Medical Center, Rochester, NY, United States

**Keywords:** respiratory pharmachology, personalised pharmacotherapy, personalised medicine, drug mechanism of action, pharmacological targets

There is an unprecedented unmet need to understand the mechanisms and therapeutic targets of pulmonary diseases. In the past, several approaches have been used to identify the respiratory disease mechanisms involving acute and chronic exposures (e.g., tobacco smoke, bacteria, viruses, hyperoxia, bleomycin) and other factors. Some targets have been successfully pinpointed based on cell signaling, kinase mechanisms, nuclear signaling, and epigenetic changes. However, these achievements have only partially translated into clinical applications.

For this Research Topic, we solicited several top-class articles on the mechanisms of acute and chronic pulmonary diseases, with a view to prioritizing the understanding of potential therapeutic targets and pharmacotherapies. Some interesting and timely topics were chosen from key authors, and a series of original articles was compiled.

Regarding the mechanisms of *in vivo* acute lung injury in experimental animal models, one study describes the role of the mitochondrial protein AKAP1 in the regulation of endoplasmic reticulum function; Sidramagowda Patil et al.; another study demonstrates the anti-inflammatory role played by metformin through its regulation of the SIRT1/nuclear factor-kB (NF-kB)/NLRP3 pathway during endothelial cell pyroptosis; Zhang et al.; and another describes the role of aspirin in reducing hyperoxia-induced pulmonary inflammation by regulating the NF-kB signaling pathway Tung et al.

In terms of potential anti-remodeling effects, one study shows that chemerin is upregulated in the lungs in acute-exposure experimental animal models *in vivo*, that it promotes the proliferation and migration of arterial smooth muscle cells *in vitro* by regulating ERK1/2 signaling, and that its plasma concentrations are elevated in persons with idiopathic pulmonary hypertension compared with healthy controls Peng et al.

Another study shows that activation of midkine-Notch2 signaling promotes the proliferation of airway smooth muscle cells *in vitro*, and in a COPD experimental animal model *in vivo*, as an airway remodeling mechanism Deng et al.

In terms of clinical studies, one article describes the development and validation of the Respiratory Adherence Care Enhancer Questionnaire and assesses its utility for identifying self-management barriers to the use of inhaled corticosteroids, the mainstay of asthma maintenance pharmacotherapy Visser et al.

Another study presents the results of a prospective, controlled, randomized clinical trial that identified the optimal dose of ropivacain, a local anesthetic, in participants who received an ultrasound-guided rhombic intercostal block (RIB) as analgesia during video-assisted thoracoscopic surgery (VATS) Deng et al.

One article summarizes recent evidence regarding the role of COVID-19 vaccines and other therapeutic interventions, focusing on emerging variants Islam et al.

One study describes the development of nanoparticle drug delivery systems actively directed against molecular targets and their potential implications for lung cancer pharmacotherapy Wang et al.

Regarding the potential pharmacological modulation of the mechanisms of allergic diseases, one *in vitro* and *in vivo* study shows that house dust mite aeroallergen increases susceptibility to pneumococcal infection by reducing leukocyte phagocytosis and NETosis Papanicolaou et al.

Finally, in the context of the emerging usage of cannabis products, one article describes the development of a standardized method for the generation of a cannabis smoke extract to investigate its mechanism of action. It also shows the inflammatory effects of cannabis smoke extract *in vitro* Aloufi et al.

We are pleased to present this series of emerging topics in this Research Topic of the Respiratory Pharmacology section of *Frontiers in Pharmacology.* These topics have high translational potential. A better understanding of the pathogenesis and mechanisms of drug action are expected to lead to identification of new therapeutic targets, which will pave the way for more personalized pharmacotherapies for respiratory diseases.

